# Tracking the Epigenetic Clock Across the Human Life Course: A Meta-analysis of Longitudinal Cohort Data

**DOI:** 10.1093/gerona/gly060

**Published:** 2018-03-20

**Authors:** Riccardo E Marioni, Matthew Suderman, Brian H Chen, Steve Horvath, Stefania Bandinelli, Tiffany Morris, Stephan Beck, Luigi Ferrucci, Nancy L Pedersen, Caroline L Relton, Ian J Deary, Sara Hägg

**Affiliations:** 1 Centre for Cognitive Ageing and Cognitive Epidemiology, University of Edinburgh, UK; 2 Medical Genetics Section, Centre for Genomic and Experimental Medicine, Institute of Genetics and Molecular Medicine, University of Edinburgh, UK; 3 Medical Research Council Integrative Epidemiology Unit, University of Bristol, UK; 4 The National Institute on Aging, NIH, Baltimore, Maryland; 5 Human Genetics, David Geffen School of Medicine, University of California Los Angeles; 6 Department of Biostatistics, School of Public Health, University of California Los Angeles; 7 Geriatric Unit, Azienda Sanitaria di Firenze, Florence, Italy; 8 UCL Cancer Institute, University College London, UK; 9 Department of Medical Epidemiology and Biostatistics, Karolinska Institutet, Stockholm, Sweden; 10 Department of Psychology, University of Edinburgh, UK

**Keywords:** Epigenetic clock, Longitudinal, Life-course perspective

## Abstract

**Background:**

Epigenetic clocks based on DNA methylation yield high correlations with chronological age in cross-sectional data. Due to a paucity of longitudinal data, it is not known how Δ_age_ (epigenetic age – chronological age) changes over time or if it remains constant from childhood to old age. Here, we investigate this using longitudinal DNA methylation data from five datasets, covering most of the human life course.

**Methods:**

Two measures of the epigenetic clock (Hannum and Horvath) are used to calculate Δ_age_ in the following cohorts: Avon Longitudinal Study of Parents and Children (ALSPAC) offspring (*n* = 986, total age-range 7–19 years, 2 waves), ALSPAC mothers (*n* = 982, 16–60 years, 2 waves), InCHIANTI (*n* = 460, 21–100 years, 2 waves), SATSA (*n* = 373, 48–99 years, 5 waves), Lothian Birth Cohort 1936 (*n* = 1,054, 70–76 years, 3 waves), and Lothian Birth Cohort 1921 (*n* = 476, 79–90 years, 3 waves). Linear mixed models were used to track longitudinal change in Δ_age_ within each cohort.

**Results:**

For both epigenetic age measures, Δ_age_ showed a declining trend in almost all of the cohorts. The correlation between Δ_age_ across waves ranged from 0.22 to 0.82 for Horvath and 0.25 to 0.71 for Hannum, with stronger associations in samples collected closer in time.

**Conclusions:**

Epigenetic age increases at a slower rate than chronological age across the life course, especially in the oldest population. Some of the effect is likely driven by survival bias, where healthy individuals are those maintained within a longitudinal study, although other factors like the age distribution of the underlying training population may also have influenced this trend.

A number of studies have demonstrated age-related methylation differences at specific CpG sites. Indeed, linear combinations of CpG methylation beta values—labelled epigenetic clocks—correlate highly with chronological age (Pearson *r* > 0.90) (1,2). For a given chronological age, older epigenetic age is presumed to indicate poorer health, and has been associated with increased mortality risk (3) and many age-related morbidities (4,5).

Because published epigenetic clocks were derived from cross-sectional data, it is unknown whether individual differences between epigenetic age and chronological age (Δ_age_) are (i) set at birth and continue unchanged over the life course, (ii) changing gradually across the life course, or (iii) changing more notably during specific periods of life, for example, adolescence and old age. Such questions can be tested using cross-sectional data, although repeated measurements at multiple times hold clear advantages for inference, especially because they are not biased by selective survival. In this study, we use longitudinal methylation data from five population-based cohorts, spanning the life course from early childhood to death.

## Methods

Longitudinal DNA methylation data were collected in five cohorts: the Avon Longitudinal Study of Parents and Children (ALSPAC), Invecchiare in Chianti (InCHIANTI), the Swedish Adoption/Twin Study of Aging (SATSA), the Lothian Birth Cohort 1936 (LBC1936), and the Lothian Birth Cohort 1921 (LBC1921).

ALSPAC is a “transgenerational prospective observational study investigating influences on health and development across the life course” (6,7). Participants comprise a cohort of offspring born to pregnant women recruited in 1991–1992 in Bristol, UK. Participants have been followed through a series of ongoing data collection waves involving questionnaires and clinical assessments. Please note that the study website contains details of all the data that is available through a fully searchable data dictionary (http://www.bris.ac.uk/alspac/researchers/data-access/data-dictionary/). DNA methylation was measured in the peripheral blood of the offspring at ages 7 and 15–17 (986 individuals corresponding to 1,901 samples) and of their mothers during pregnancy and approximately 18 years later (982 individuals corresponding to 1,816 samples). The resulting profiles form part of the Accessible Resource for Integrated Epigenomics Studies (ARIES) dataset (8). Data are available by request from the Avon Longitudinal Study of Parents and Children Executive Committee (http://www.bristol.ac.uk/alspac/researchers/access/).

The InCHIANTI study is a population-based prospective cohort study of residents aged 20 or older from two areas in the Chianti region of Tuscany, Italy. Sampling and data collection procedures have been described elsewhere (9). Briefly, 1,326 participants donated a blood sample at baseline (1998–2000), of which 784 also donated a blood sample at the 9-year follow-up (2007–2009). DNA methylation was assayed in participants with sufficient DNA at both baseline and 9-year visits (*n* = 499). After samples and data quality checks, DNA methylation data at baseline and follow-up were available in 460 individuals.

The SATSA study is a longitudinal prospective cohort study of adult Swedish twins (10,11). It was started in 1984 and has been ongoing until 2014. There are up to 10 waves of in-person testing available with questionnaire data on health and life-style choices, cognitive testing, physical performance measures, anthropometrics, and blood draws. DNA methylation was assessed repeatedly up to five times in 373 individuals corresponding to 938 samples. The age ranges in the methylation SATSA samples spanned from 48 to 89 years at baseline (1992) and 63 to 99 years at the last follow-up (2012).

LBC1921 and LBC1936 are birth cohorts containing Scottish participants born in 1921 and 1936, most of whom participated in the Scottish Mental Surveys of 1932 and 1947, when nearly all 11-year old Scottish children completed a cognitive test (12,13). Longitudinal follow up of those living in the Lothian area began in 2000 for those born in 1921 and in 2006 for those born in 1936. Blood-based DNA methylation data were available at three times in both cohorts—mean ages 70, 73, and 76 years in LBC1936 (1,054 individuals corresponding to 2,338 samples), and mean ages 79, 87, and 90 in LBC1921 (476 individuals corresponding to 703 samples).

### Ethics

In ALSPAC, informed written consent was obtained from parents of participants after receiving a complete description of the study at the time of enrolment into the ALSPAC project, with the option for them or their children to withdraw at any time. Ethical approval for the ALSPAC study was obtained from the ALSPAC Ethics and Law Committee and the Local Research Ethics Committees.

InCHIANTI participants provided written informed consent to participate in this study. The study complied with the Declaration of Helsinki. The Italian National Institute of Research and Care on Aging Institutional Review Board approved the study protocol and study participants provided informed consent.

Participants in SATSA provided informed consents at each testing occasion. The longitudinal collection and analyses of data have been approved at several occasions by the Research Ethics Committee at Karolinska Institutet with Dnrs 84:61, and 98–319, and by the Regional Ethics Board in Stockholm with Dnrs 2007/151-31/4, 2010/657-31/3, and 2015/1729–31/5.

Following written informed consent, venesected whole blood was collected for DNA extraction in both LBC1921 and LBC1936. Ethics permission for the LBC1921 was obtained from the Lothian Research Ethics Committee (Wave 1: LREC/1998/4/183). Ethics permission for the LBC1936 was obtained from the Multi-Centre Research Ethics Committee for Scotland (Wave 1: MREC/01/0/56), the Lothian Research Ethics Committee (Wave 1: LREC/2003/2/29), and the Scotland A Research Ethics Committee (Waves 2 and 3: 07/MRE00/58).

### DNA Methylation and Epigenetic Clock Measurements

Blood-based Illumina 450k methylation data were obtained separately in each cohort. Cohort specific quality control details have been reported previously (14–17). Epigenetic age was calculated by multiplying beta values by the regression weights from Horvath (2) and Hannum *et al.* (1) to create the respective clocks. The Hannum clock was derived using DNA methylation from blood in a single cohort of 656 individuals; the Horvath clock was derived using DNA methylation from 51 tissue types across 8,000 individuals from multiple studies. The Hannum clock was developed from the Illumina 450K array, while the Horvath clock was restricted to approximately 21,000 probes common to both the Illumina 27K and 450K arrays. Further, the Hannum clock is moderately correlated with proportions of certain blood cells, while the Horvath clock is relatively uncorrelated with blood cell counts to date. Delta age was defined as a simple subtraction of chronological age from epigenetic age using both versions of the clock. Cell count predictions were estimated from DNA methylation data using the Houseman method in all cohorts (18).

### Statistical Analysis

Linear mixed models were used to assess longitudinal change in Δ_age_ separately in each cohort. Δ_age_ was modelled as the outcome, with chronological age as the time-scale and predictor of interest. All models controlled for sex and a random effect intercept term. The SATSA study further adjusted for the twin structure in the data by allowing for additional random effects within twin pairs. Analyses were conducted in R using the lme4 and lmerTest packages. Pearson correlations were calculated for Horvath and Hannum Δ_age_ between waves for all pair-wise combinations in each cohort. Sensitivity analyses were carried out using only individuals present in at least three waves and using cell count prediction adjustments for the Hannum clock.

## Results

The total number of individuals participating in this study was 4,075 with a total of 8,616 samples. Summary statistics of chronologic and epigenetic age at each study wave of the participating cohorts are presented in Table 1. Individuals from the different cohorts represent most of the human life course, from young children to the oldest old, although the majority of the samples were drawn in later life. All cohorts with the exception of the ALSPAC mothers had an even distribution of men and women. The number of follow-up occasions in the cohorts varied from two to five, with different time intervals in between the measurements.

**Table 1.** Characteristics of Longitudinal Methylation Cohorts

Cohort	Wave	Year	Participants (*N*)	Women (%)	Age mean ± *SD*	Horvath Age Mean ± *SD*	Hannum Age Mean ± *SD*
SATSA	1	1992–1994	212	60	68.3 ± 9.1	60.3 ± 9.9	65.3 ± 9.3
	2	1999–2001	227	63	71.0 ± 10.1	63.2 ± 8.8	66.9 ± 8.9
	3	2002–2004	178	54	72.1 ± 9.2	64.0 ± 8.8	68.3 ± 9.1
	4	2008–2010	172	61	75.9 ± 8.2	67.3 ± 9.2	71.0 ± 7.8
	5	2010–2012	149	66	77.8 ± 8.2	67.3 ± 9.1	71.4 ± 7.8
LBC1936	1	2004–2007	920	49	69.6 ± 0.8	66.0 ± 6.5	71.3 ± 5.8
	2	2007–2010	800	48	72.5 ± 0.7	69.3 ± 6.6	72.9 ± 5.7
	3	2011–2013	618	48	76.3 ± 0.7	72.6 ± 6.4	77.6 ± 5.6
LBC1921	1	1999–2001	446	60	79.1 ± 0.6	73.7 ± 7.0	80.3 ± 6.2
	2	2007–2008	175	54	86.7 ± 0.4	77.6 ± 6.0	81.6 ± 6.2
	3	2011–2012	82	54	90.1 ± 0.9	79.3 ± 6.1	84.8 ± 5.8
ALSPAC	1	1998–2000	948	50	7.5 ± 0.15	8.3 ± 2.4	9.2 ± 4.6
Children	2	2006–2010	953	52	17.1 ± 1.0	17.2 ± 4.3	20.4 ± 4.9
ALSPAC	1	1991–1992	924	100	29.1 ± 4.4	30.2 ± 6.7	34.9 ± 5.6
Mothers	2	2008–2011	892	100	47.4 ± 4.5	45.1 ± 6.5	47.1 ± 6.2
InCHIANTI	1	1998–2000	460	54	62.2 ± 16.1	61.6 ± 13.3	67.6 ± 16.0
	2	2007–2009	460	54	71.4 ± 16.2	68.7 ± 13.1	75.2 ± 15.9

**Figure 1.**
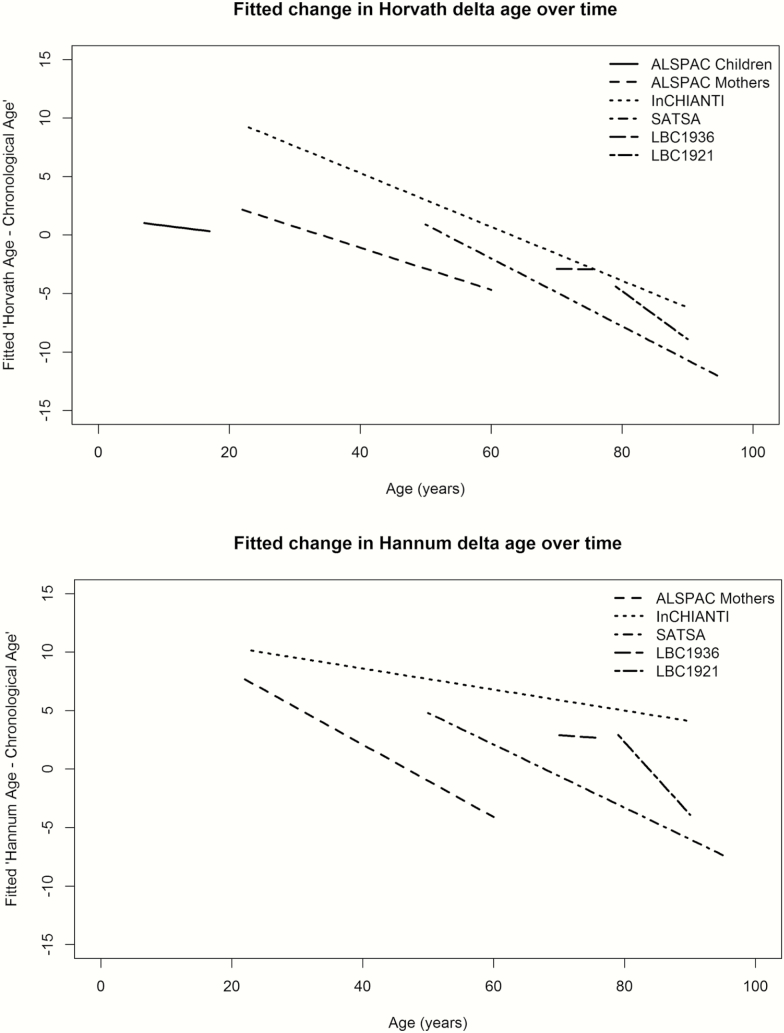
Mean linear longitudinal trajectories of epigenetic Δ_age_. For each data set, mixed models were applied and predicted values, derived from the model intercept and fixed effect estimates for age, were plotted to illustrate the Δ_age_ trajectories across the life span. The *x*-axis represents the age where the cohort specific trajectory is plotted corresponding to the age span covered in that cohort. The *y*-axis shows the Δ_age_.

The fitted mean trajectories of Δ_age_ over time for each cohort are presented together in Figure 1. As the Hannum clock was not trained on children, the ALSPAC children were excluded from the Hannum plot. Participant level trajectories for each cohort for the Horvath- and Hannum epigenetic clock measures, as well as respective Δ_age_, are shown in Supplementary Figure 1A–D. Judging from the trajectories and the mixed model output (Supplementary Table 1), Δ_age_ declines during adulthood (β_Horvath_ ranged from −0.18 to −0.40, all *P* < 2 × 10^−16^; β_Hannum_ ranged from −0.09 to −0.62, all *P* < 4 × 10^−16^), and also during childhood, although to a smaller degree (β_Horvath_ = −0.07, *P* = 8 × 10^−7^). In other words, over time, the DNA methylation-based biological clock increases at a slower rate than chronological age. The only exception to this trend was the Lothian Birth Cohort 1936, where Δ_age_ remained constant over a 6-year interval between ages 70 and 76 years (β_Horvath_ = −0.01, *P* = 0.81; β_Hannum_ = −0.04, *p* = .16). Sensitivity analyses keeping only individuals with at least three waves of measurements (*n* = 183 in SATSA, 487 in LBC1936, and 66 in LBC1921) did not change the results (Supplementary Table 2). Likewise, adjustments for predicted cell counts for the Hannum epigenetic clock measurements did not change the age estimates remarkably (Supplementary Table 3).

**Figure 2.**
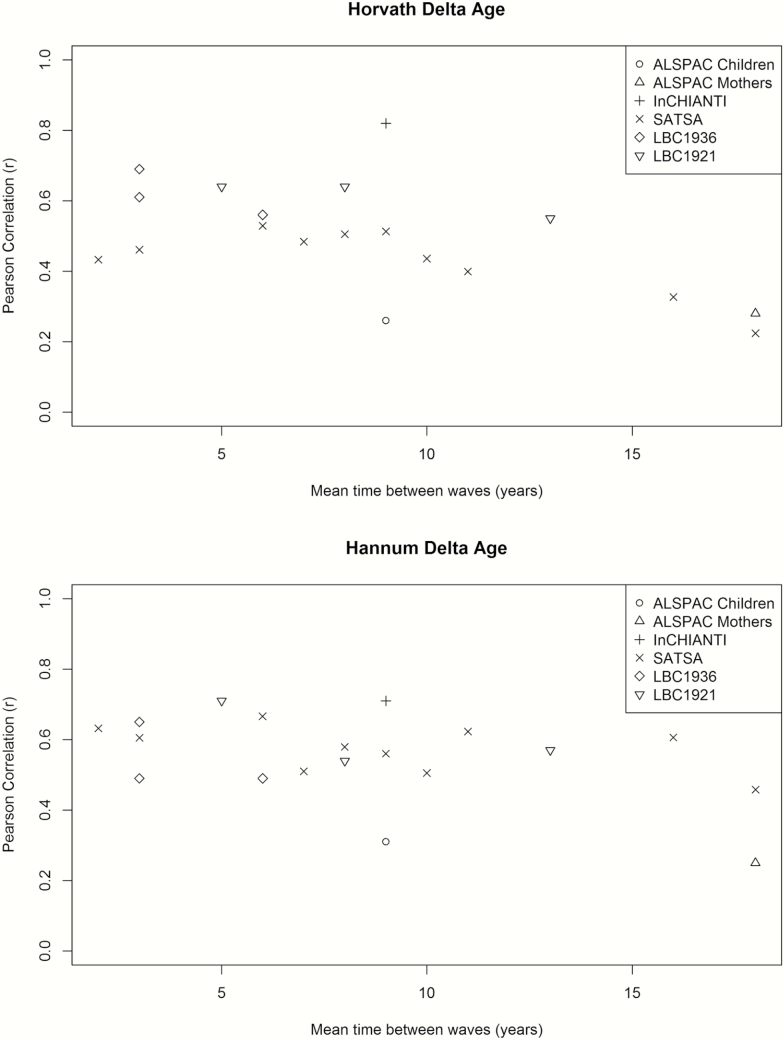
Within-cohort correlations of epigenetic Δ_age_ across study waves. For each data set, correlations between all possible combinations of waves were calculated and plotted. On the *x*-axis is the time between the two measurements in years and on the *y*-axis is the correlation coefficient.

The correlation between Δ_age_ across waves is presented by cohort in Table 2, and ranged from 0.22 to 0.82 for Horvath and 0.25 to 0.71 for Hannum. The association of between-wave correlations and sampling times between the waves is illustrated in Figure 2, where increasing sampling time confers a lower correlation between samples for Horvath Δ_age_ (Beta=-0.015 units per year, *p*-value = 9.3 × 10^−4^), but less so for Hannum Δ_age_ (Beta=-0.009 units per year, *p*-value = .039). Sensitivity analyses keeping only individuals with three measures confirmed the decreasing correlation pattern for Horvath Δ_age_ (Beta=-0.016 units per year, *p*-value = 2.5 × 10^−4^) but not for Hannum Δ_age_ (Beta = −0.005 units per year, *p*-value = .28).

**Table 2.**
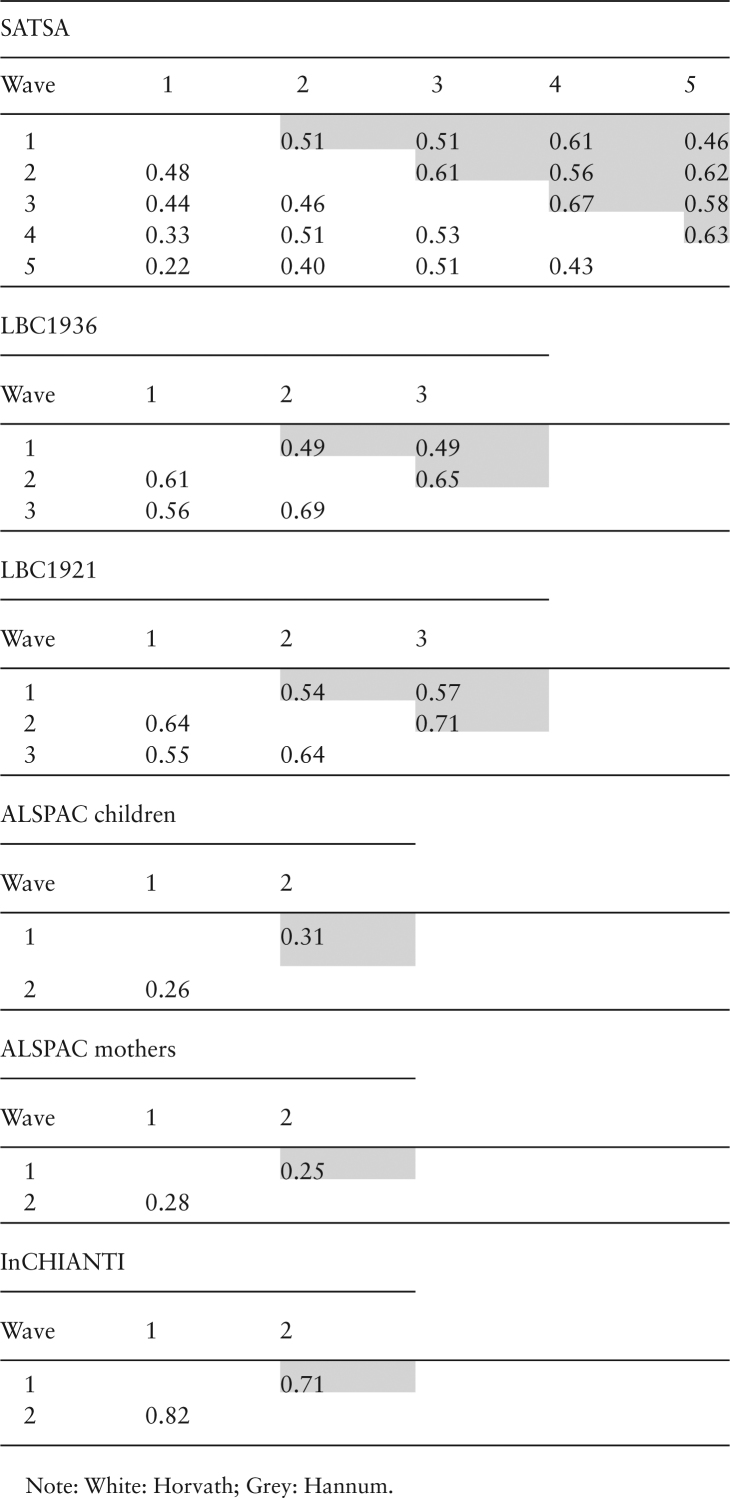
Pearson Correlations of Epigenetic Δ_age_ Between Waves in Each Cohort

## Discussion

In this article, we presented the first comprehensive analysis of the epigenetic clock from a longitudinal perspective by analysing data from five prospective cohorts with repeated sampling. We showed that epigenetic age was highly correlated with chronologic age when including multiple samples per individual, and that Δ_age_ declined over the life span. Moreover, cross-correlations of Δ_age_ from different waves indicated more similar patterns in samples collected closer in time compared to samples collected further apart, although the pattern was more prominent in Horvath than in Hannum estimates.

The epigenetic clock has been shown to be a useful marker of biological age when using data from cross-sectional sample collections (19). Here, we provided evidence for its usefulness in a longitudinal perspective. The overall correlations with chronologic age were high, as judged from the individual trajectory plots, and thus comparable to cross-sectional study findings. However, the trajectories of Δ_age_ showed a declining trend in almost all of the cohorts with adult sample collections. This indicates that epigenetic age increases at a slower rate than chronological age, especially in the oldest population. Some of the effect is likely driven by survival bias, where healthy individuals are those maintained within a longitudinal study, although other factors like underlying training population for the respective clocks may also have influenced this trend. It may also be possible that there is a ceiling effect for Δ_age_ whereby epigenetic clock estimates plateau. In children, the Horvath epigenetic age declined with chronologic age, although less so than in the adult life span, while the Hannum clock was not trained in children and hence was not used.

The investigation of correlations between different waves in each cohort showed a decreasing correlation for samples collected further apart, which is in line with expectations where measures further apart have been more influenced by other (environmental) factors. However, it should be noted that the trend is somewhat different for Horvath and Hannum clocks. This is perhaps not surprising given that they represent different tissues; Horvath is a multi-tissue clock built to capture more variation while Hannum only applies to blood leukocytes (only a few CpG sites overlap in the two clocks) and all our samples came from blood leukocytes.

The strength of this study is the joint effort of combining five longitudinal cohorts, comprising six data sets, with repeated sample collections assessed by DNA methylation 450k arrays. By doing so, we were able to capture the full life-course perspective of the epigenetic clock from childhood to old age. However, there is an overrepresentation of samples collected at the later part of the life span, which limits the interpretations. Moreover, these cohorts are all based on individuals from a European ancestry background. As there is evidence for differences based on the epigenetic clock in other ethnic populations (20), our findings are not necessarily generalizable to other ethnicities.

In summary, we have provided an analysis of longitudinal trajectories of the epigenetic clock across the life course, showing that epigenetic age increases at a slower rate than chronological age across the life course, especially in the oldest population.

## Funding

The UK Medical Research Council and Wellcome (grant number 102215/2/13/2) and the University of Bristol provide core support for ALSPAC. This publication is the work of the authors and Matthew Suderman will serve as guarantor for the ALSPAC-related contents of this article. Analysis of the ALSPAC data was funded by UK Economic and Social Research Council grant (grant number ES/N000498/1). ARIES was funded by the BBSRC (BBI025751/1 and BB/I025263/1). Supplementary funding to generate DNA methylation data which are (or will be) included in ARIES has been obtained from the MRC, ESRC, NIH, and other sources. ARIES is maintained under the auspices of the MRC Integrative Epidemiology Unit at the University of Bristol (grant numbers MC_UU_12013/2, MC_UU_12013/8 and MC_UU_12013/9). The InCHIANTI study baseline (1998–2000) was supported as a “targeted project” (ICS110.1/RF97.71) by the Italian Ministry of Health and in part by the U.S. National Institute on Aging (Contracts: 263 MD 9164 and 263 MD 821336). The SATSA study was supported by NIH grants R01 AG04563, AG10175, AG028555, the MacArthur Foundation Research Network on Successful Aging, the Swedish Council for Working Life and Social Research (FAS/FORTE) (97:0147:1B, 2009-0795, 2013–2292), the Swedish Research Council (825-2007-7460, 825-2009-6141, 521-2013-8689, 2015–03255), KI Foundation, the Strategic Research Program in Epidemiology at Karolinska Institutet, and by Erik Rönnbergs donation for scientific studies in aging and age-related diseases.

R.E.M. and I.J.D. conducted the research in The University of Edinburgh Centre for Cognitive Ageing and Cognitive Epidemiology (CCACE), part of the cross-council Lifelong Health and Wellbeing Initiative (MR/K026992/1); funding from the Biotechnology and Biological Sciences Research Council (BBSRC) and Medical Research Council (MRC) is gratefully acknowledged. Phenotype collection in the Lothian Birth Cohort 1921 was supported by the UK’s Biotechnology and Biological Sciences Research Council (BBSRC), The Royal Society and The Chief Scientist Office of the Scottish Government. Phenotype collection in the Lothian Birth Cohort 1936 was supported by Age UK (The Disconnected Mind project). Methylation typing was supported by Centre for Cognitive Ageing and Cognitive Epidemiology (Pilot Fund award), Age UK, The Wellcome Trust Institutional Strategic Support Fund, The University of Edinburgh, and The University of Queensland.

## Conflict of interest statement

None declared.

## Supplementary Material

Supplementary Data
