# Supplementary material for: Tracking the Epigenetic Clock Across the Human Life Course: A Meta-analysis of Longitudinal Cohort Data
Source: J Gerontol A Biol Sci Med Sci. 2018 Mar 20;74(1):57–61. doi: 10.1093/gerona/gly060 (PMC6298183; doi:10.1093/gerona/gly060)
Supplement: Supplementary Data [file gly060_suppl_supplementary_data.docx]

**Supplementary Data**

**Tracking the epigenetic clock across the human life-course - a meta-analysis of longitudinal cohort data**

Riccardo E. Marioni^1,2^, Matthew Suderman^3^, Brian H. Chen^4^, Steve Horvath^5,6^, Stefania Bandinelli^7^, Tiffany Morris^8^, Stephan Beck^8^, Luigi Ferrucci^4^, Nancy L. Pedersen^9^, Caroline L. Relton^3^, Ian J. Deary^1,10^, and Sara Hägg^9^

**Supplementary Figure 1.** Cohort-specific individual trajectories of epigenetic age. Plots of individual trajectories where each line is representing one individual across the longitudinal study period is presented for **A)** ALSPAC, **B)** InCHIANTI, **C)** SATSA, and **D)** LBC. The panels on the right are Horvath estimates and on the left Hannum. Upper panel is epigenetic age and lower panel is Δ_age_. Thick red lines represent the perfect correlation while the blue lines are the average regression line.

**A)** The Avon Longitudinal Study of Parents and Children (ALSPAC)


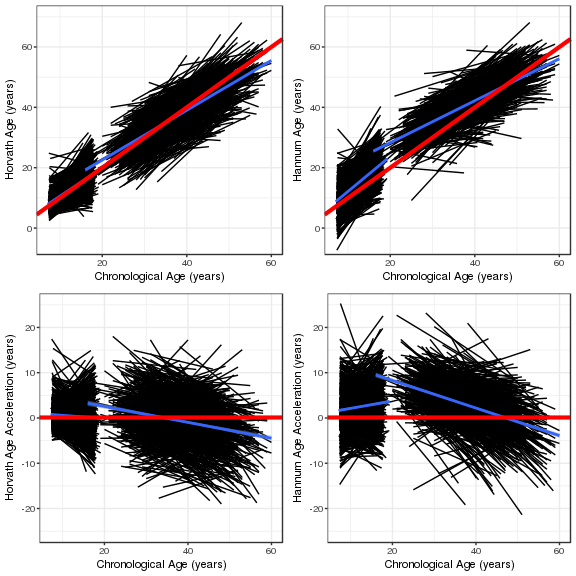


**B)** Invecchiare in Chianti (InCHIANTI)

**C)** The Swedish Adoption/Twin Study of Aging (SATSA)


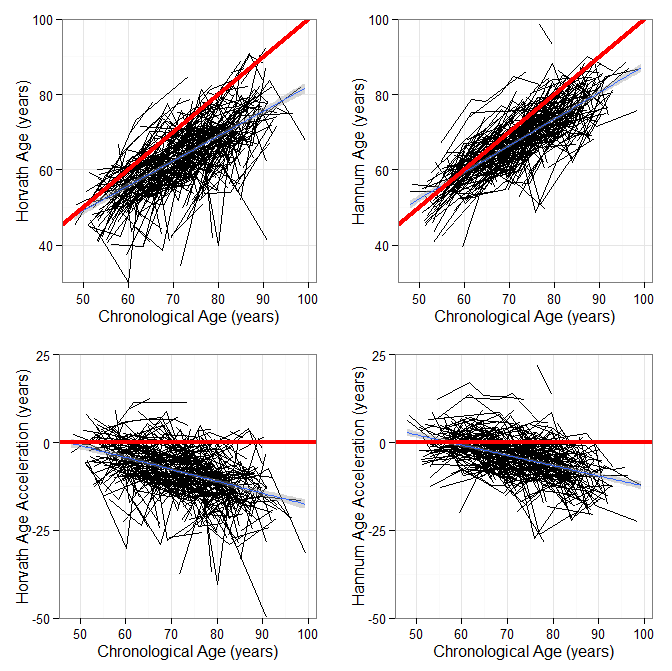


**D)** Lothian Birth Cohorts 1921 and 1936


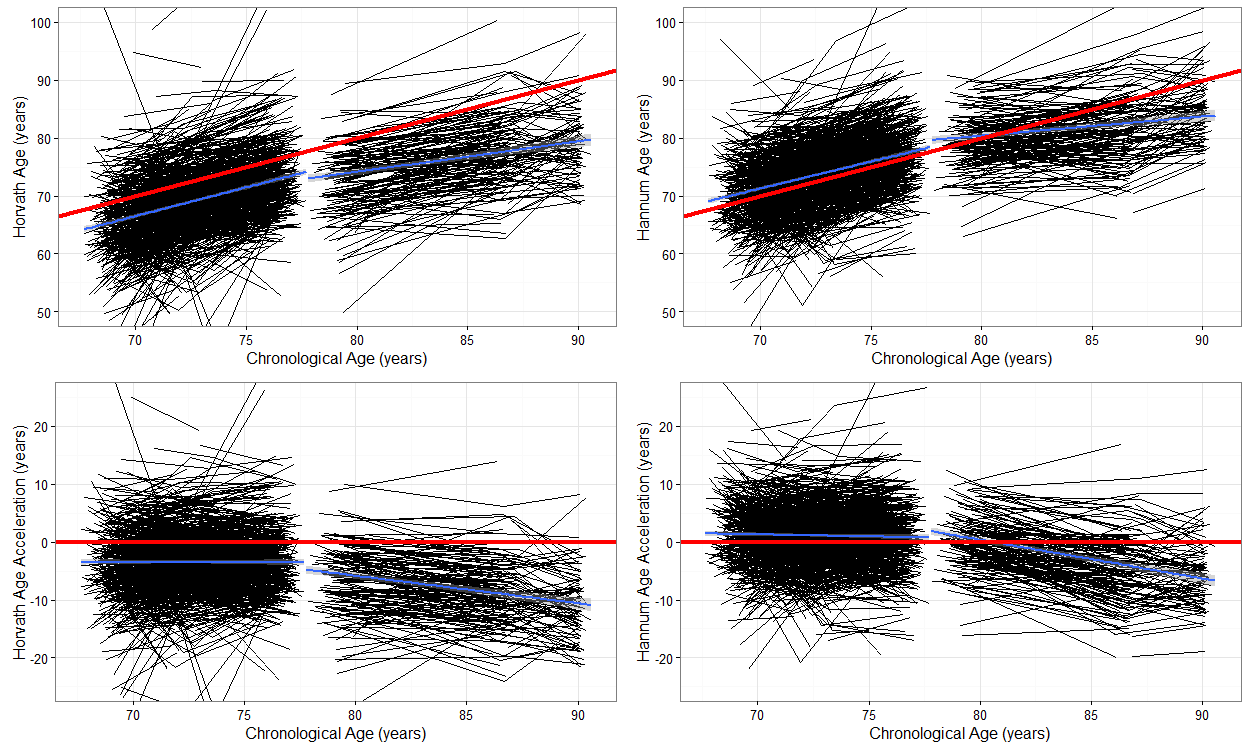


**Supplementary Table 1.** Linear mixed model output for change in Δ_age_ over time

| **Cohort** | **Epigenetic clock** | **β_age_** | **SE** | **P** |
| --- | --- | --- | --- | --- |
| ALSPAC children | Horvath | -0.07 | 0.01 | 8x10-7 |
| ALSPAC mothers | Horvath | -0.18 | 0.01 | <2x10-16 |
| InCHIANTI | Horvath | -0.23 | 0.01 | <2x10-16 |
| SATSA | Horvath | -0.29 | 0.02 | <2x10-16 |
| LBC1936 | Horvath | -0.01 | 0.03 | 0.81 |
| LBC1921 | Horvath | -0.40 | 0.04 | <2x10-16 |
|  |  |  |  |  |
| ALSPAC children | Hannum | - | - | - |
| ALSPAC mothers | Hannum | -0.31 | 0.01 | <2x10-16 |
| InCHIANTI | Hannum | -0.09 | 0.01 | 4x10-16 |
| SATSA | Hannum | -0.25 | 0.02 | <2x10-16 |
| LBC1936 | Hannum | -0.04 | 0.03 | 0.16 |
| LBC1921 | Hannum | -0.62 | 0.04 | <2x10-16 |

**Supplementary Table 2.** Linear mixed model output for change in Δ_age_ over time across individuals with at least three time points

| **Cohort** | **Epigenetic clock** | **β_age_** | **SE** | **P** |
| --- | --- | --- | --- | --- |
| SATSA | Horvath | -0.29 | 0.03 | <2x10-16 |
| LBC1936 | Horvath | 0.01 | 0.04 | 0.77 |
| LBC1921 | Horvath | -0.40 | 0.06 | 6.0x10-10 |
|  |  |  |  |  |
| SATSA | Hannum | -0.24 | 0.02 | <2x10-16 |
| LBC1936 | Hannum | -0.006 | 0.04 | 0.87 |
| LBC1921 | Hannum | -0.51 | 0.05 | <2x10-16 |

**Supplementary Table 3.** Linear mixed model output for change in Δ_age_ over time adjusted for predicted cell counts in blood samples (Hannum only)

| **Cohort** | **β_age_** | **SE** | **P** |
| --- | --- | --- | --- |
| ALSPAC mothers | -0.18 | 0.01 | <2x10-16 |
| InCHIANTI | -0.11 | 0.01 | <2x10-16 |
| SATSA | -0.27 | 0.02 | <2x10-16 |
| LBC1936 | 0.10 | 0.03 | 0.001 |
| LBC1921 | -0.51 | 0.04 | <2x10-16 |

**Supplementary Table 4**. Pearson correlations of Δ_age_ between waves in individuals with at least three time points

| SATSA |  | | |  |  |
| --- | --- | --- | --- | --- | --- |
| Wave | 1 | 2 | 3 | 4 | 5 |
| 1 |  | 0.45 | 0.53 | 0.61 | 0.46 |
| 2 | 0.51 |  | 0.58 | 0.54 | 0.62 |
| 3 | 0.42 | 0.53 |  | 0.66 | 0.59 |
| 4 | 0.31 | 0.49 | 0.49 |  | 0.66 |
| 5 | 0.22 | 0.40 | 0.50 | 0.44 |  |
|  |  |  |  |  |  |
| LBC1936 |  |  |  |  |  |
| Wave | 1 | 2 | 3 |  |  |
| 1 |  | 0.48 | 0.48 |  |  |
| 2 | 0.58 |  | 0.65 |  |  |
| 3 | 0.55 | 0.68 |  |  |  |
|  |  |  | |  |  |
| LBC1921 |  | | |  |  |
| Wave | 1 | 2 | 3 |  |  |
| 1 |  | 0.60 | 0.61 |  |  |
| 2 | 0.60 |  | 0.67 |  |  |
| 3 | 0.59 | 0.65 |  |  |  |

White: Horvath; Grey: Hannum

**Supplementary Table 5**. Spearman rank correlations of Δ_age_ between waves in individuals with at least three time points

| SATSA |  | | |  |  |
| --- | --- | --- | --- | --- | --- |
| Wave | 1 | 2 | 3 | 4 | 5 |
| 1 |  | 0.40 | 0.53 | 0.52 | 0.46 |
| 2 | 0.54 |  | 0.54 | 0.50 | 0.64 |
| 3 | 0.44 | 0.59 |  | 0.52 | 0.53 |
| 4 | 0.32 | 0.56 | 0.51 |  | 0.59 |
| 5 | 0.26 | 0.39 | 0.56 | 0.53 |  |
|  |  |  |  |  |  |
| LBC1936 |  |  |  |  |  |
| Wave | 1 | 2 | 3 |  |  |
| 1 |  | 0.51 | 0.51 |  |  |
| 2 | 0.56 |  | 0.64 |  |  |
| 3 | 0.62 | 0.72 |  |  |  |
|  |  |  | |  |  |
| LBC1921 |  | | |  |  |
| Wave | 1 | 2 | 3 |  |  |
| 1 |  | 0.60 | 0.62 |  |  |
| 2 | 0.61 |  | 0.62 |  |  |
| 3 | 0.62 | 0.60 |  |  |  |

White: Horvath; Grey: Hannum
